# Nutrition-Related Knowledge, Attitudes, and Practices (KAP) among Kindergarten Teachers in Chongqing, China: A Cross-Sectional Survey

**DOI:** 10.3390/ijerph15040615

**Published:** 2018-03-28

**Authors:** Hongyan Liu, Xianglong Xu, Dengyuan Liu, Yunshuang Rao, Cesar Reis, Manoj Sharma, Jun Yuan, Yao Chen, Yong Zhao

**Affiliations:** 1 School of Public Health and Management, Chongqing Medical University, No. 1 Yixueyuan Road, Yuzhong District, Chongqing 400016, China; 15178844252@163.com (H.L.); xianglong1989@126.com (X.X.); dengyuanliu@foxmail.com (D.L.); rys0606@163.com (Y.R.); yuanjun@cqmu.edu.cn (J.Y.); 2 Research Center for Medicine and Social Development, Chongqing Medical University, Chongqing 400016, China; 3 Collaborative Innovation Center of Social Risks Governance in Health, Chongqing Medical University, Chongqing 400016, China; 4 Preventive Medicine Department, Loma Linda University Medical Center, Loma Linda, CA 92354, USA; cesarreis@hotmail.com; 5 Behavioral & Environmental Health, School of Public Health, Jackson State University, 350 West Woodrow Wilson Avenue, Jackson, MS 39213, USA; manoj.sharma@jsums.edu; 6 Health Sciences, Walden University, 100 Washington Avenue South, Suite 900, Minneapolis, MN 55401, USA; 7 Medical Examination Center, Second Affiliated Hospital of Chongqing Medical University, Chongqing 400016, China; chenyao11526@aliyun.com

**Keywords:** kindergarten teachers, nutrition, knowledge, attitude, behavior, China

## Abstract

Kindergarten teachers play an important role in providing kindergarten children with education on nutrition. However, few studies have been published on nutrition-related knowledge, attitudes, and practices (KAP) of Chinese kindergarten teachers. This study aimed to assess the nutrition-related knowledge, attitudes, and practices (KAP) of kindergarten teachers in Chongqing, China. Thus, a cross-sectional survey was conducted using a structured KAP model questionnaire administered to 222 kindergarten teachers, who were senior teachers from 80 kindergartens in 19 districts and 20 counties in Chongqing. Multiple regression analysis was used to analyze the influential factors. Among the participants, 54.2% were familiar with simple nutrition-related knowledge; only 9.9% of them were satisfied with their knowledge of childhood nutrition; and 97.7% of them had a positive attitude to learn nutrition-related knowledge. Only 38.7% of the participants had attended pediatric nutrition knowledge courses or training. Multiple regression analysis confirmed significant independent effects on the nutrition knowledge score (*p* < 0.0001) of respondents on age, type of residence, type of kindergarten, body mass index(BMI), professional training of kindergarten teachers, behavior of having ever participated in childhood nutrition education knowledge courses or training, and behavior of having ever paid attention to children’s nutrition knowledge. The model indicated that independent variables explained 45.4% (adjusted R^2^) of the variance found in the knowledge scores of respondents. While there were low levels of nutrition knowledge and training, it was still encouraging to note that there were positive attitudes towards acquiring nutrition-related knowledge among kindergarten teachers in Chongqing, China. These findings provide some implications that necessary training measures need to be carried out to improve the nutrition-related knowledge level among kindergarten teachers in China.

## 1. Introduction

Being overweight and obese among preschool children is a major public health issue. In 2016, 41 million preschool children were overweight globally [1]. In developed countries, the prevalence rate of obese and overweight children aged up to five years increased from 7.9% in 1990 to 11.7% in 2010; this rate is expected to reach 14.1% in 2020 [2]. In the United States, the prevalence rate of obesity among children aged two to five years is 17.0% and extreme obesity was 5.8% in 2011–2014 [3]. In China, the prevalence of overweight and obese children under six years of age in 2012 was 8.4% and 3.1%, respectively, which increased by 1.9% and 0.4%, respectively, from the figures in 2002 [4]. The importance of decreasing the prevalence of obesity among children was emphasized by a study that showed that childhood obesity adversely affected nearly every organ system and caused serious health consequences [5].

Solving the nutritional health problems among kindergarten children is an urgent unmet need. According to the National Statistical Bulletin on the Development of Education in 2016, there were 239,800 kindergartens in China, which increased 16,100 over the last year; there were 44,138,600 preschool children in China, which increased 1,490,300 over the last year; there were 2,498,800 kindergarten principals and teachers in China, which increased 195,600 over the last year [6]. Studies have shown that preschool children spend considerable time at preschool [7]. Previous studies demonstrated that insufficient or excessive nutrient supply, unreasonable nutritional allocation, picky eating among a large proportion of preschool children, unsound dietary nutrition management, and a lack of nutrition knowledge among teachers and staff are common in the kindergartens [8,9,10,11]. Due to the increase in the number of children in kindergartens and their nutritional health problems, the nutritional health education at schools becomes key to solving the nutrition and health problems of children. Some studies have shown that an effective way to reduce health consequences is to establish and promote healthy eating behaviors early, starting with utilization of the educational system [12,13,14,15,16].

The health behaviors of kindergarten teachers may have a far-reaching effect on the general health behaviors and future lives of kindergarten children [17]. Health-related roles of kindergarten teachers are as follows: providing first aid [18], fostering self-regulation among kindergarten children [19], assuming increased responsibility and initiatives for children’s healthy habits [20], enhancing young children’s motivation to learn [21], and recognizing health problems and allocating children to suitable early prevention schemes [17]. A study showed that delivering nutrition education through the classroom teacher gains greater acceptance among children rather than coming through an outside nutrition expert [22]. Nutrition education for children was mainly handled by kindergarten teachers in China, especially in Chongqing, because there were almost no registered dietitians and other nutrition education professionals in kindergartens. 

Descriptive studies on nutrition-related knowledge of kindergarten teachers demonstrated that these teachers possess only a limited amount of nutrition-related knowledge [23,24,25]. Furthermore, little is known about factors that affect nutrition-related knowledge among the Chinese teachers. Therefore, in view of the critical role of nutritional education of kindergarten teachers to kindergarten children, the present study aimed to assess nutrition-related knowledge, attitudes, and practices (KAP) among these teachers in Chongqing, China.

## 2. Materials and Methods

### 2.1. Ethical Approval

The study protocol was approved by the Ethics Committee, Chongqing Medical University (Preference number: 2016001). Written informed consent was obtained from all participants.

### 2.2. Study Design

A cross-sectional survey was conducted from November 2016 to November 2017 in Chongqing, China. Multi-stage stratified sampling was applied to select 80 kindergartens (selecting 2 to 3 teachers in each kindergarten) from 19 districts and 20 counties in Chongqing, China. Interviewees were senior kindergarten teachers. A self-administered questionnaire was given to all respondents after obtaining their informed consents. A total of 226 kindergarten teachers participated in this study. Almost all preschool teachers (*n* = 226, 94.16% response rate) completed the survey, and 4 questionnaires were excluded because of missing data, resulting in a final sample of 222 for analysis. No monetary incentive or material award was provided for participation.

### 2.3. Questionnaires

A self-designed questionnaire was used in this study based on the KAP model and Chinese Dietary Guide (DRIs). The questionnaire included four sections:

(1) Demographic characteristics

Socioeconomic characteristics: Age (17–30 years old/31–40 years old/≥41 years old), gender (male/female), marital status (married/unmarried), residence (rural/urban), and educational level (≤senior middle school–basic education/vocational, technical secondary school and junior college–secondary education/≥senior college and higher than university education level–higher education).

Kindergarten teachers’ specific questions: type of kindergarten (public/private kindergarten), working lifetime (<3 years/3–10 years/>10 years), and professional training (yes/no).

BMI (Body mass index): Participants were classified using the U.S. Centers for Disease Control and Prevention [26]: underweight (BMI < 18.5), normal weight (18.5 ≤ BMI < 25), overweight (25 ≤ BMI < 30), and obesity (BMI ≥ 30). We merged the obesity BMI group with overweight BMI group into a single group because of the finite participants of the obesity BMI group. Weight and height obtained in the survey were all self-reported by the respondents.

(2) Nutrition-related knowledge 

A nutrition-related knowledge questionnaire that contained seven parts and 53 questions was used to comprehensively assess the level of kindergarten teachers’ nutritional knowledge. Topics, such as protein-related knowledge (7 items), fat-related knowledge (7 items), vitamin-related knowledge (13 items), calcium-related knowledge (4 items), dietary fiber-related knowledge (9 items), nutrient elements-related knowledge (3 items), and children’s nutrition-related knowledge (10 items), were asked through a total of 53 questions (see Supplementary Materials S1). The score of nutrition-related knowledge was calculated using the assignment method. Each question can be directly judged as true or false. Correct answers received a score of 1 and incorrect answers received a score of 0. The total possible score of this section was 53 points. 

(3) Nutrition knowledge learning-related attitudes

The following five questionnaire items measured nutrition knowledge-related attitudes among kindergarten teachers: “Do you have the confidence to do the childhood nutrition work well (confidence/neutral/no confidence)?”, “Are you willing to learn more knowledge about children-related nutrition (willing/neutral/unwilling)?”, “Are you satisfied with the childhood nutrition knowledge you already have (satisfied/neutral/unsatisfied)?”, “Are you willing to attend more young childhood nutrition education and training (willing/neutral/unwilling)?”, and “Are you willing to get information related to children’s nutrition knowledge through new media (micro blog, WeChat, QQ, and others) (willing/neutral/unwilling)?”

(4) Nutrition knowledge learning-related practices or behaviors

The following four questionnaire items measured nutrition knowledge-related practices or behaviors among kindergarten teachers: “Have you ever participated in childhood nutrition education knowledge courses or training (yes/no)?”, “Have you ever paid attention to children’s nutrition knowledge (yes/no)?”, “Have you ever taken the initiative to promote children’s nutrition knowledge to your relatives or friends (never/sometimes/often/always)?”, and “Have you ever taken the initiative to learn about child nutrition in your spare time (never/sometimes/often/always)?”.

### 2.4. Internal Validity

In the final questionnaire, the reliability of the nutrition-related knowledge section among 222 kindergarten teachers in Chongqing, China was evaluated by assessing the internal reliability and the Cronbach’s α was 0.801. The reliability of three sections, including the nutrition-related knowledge, nutrition knowledge-related attitudes, and nutrition knowledge-related practices or behaviors, among 191 kindergarten teachers (31 teachers were excluded because of missing data) in Chongqing, China, was evaluated by assessing the internal reliability and the Cronbach’s α was 0.737.

### 2.5. Data Analysis

IBM SPSS 23.0 software (SPSS Inc., Chicago, IL, USA) was used to analyze data. Invalid data or missing data were excluded, and all data entries were double-checked to prevent errors. Descriptive statistics, including frequency and percentage, were calculated on demographic characteristics of survey respondents. *T*-tests, ANOVA, and Mann–Whitney U tests were employed to compare differences among continuous variables. Multiple linear regression models and enter methods were performed to assess factors that affect nutrition-related knowledge. Variables with statistical significance in single factor analysis were included in multiple linear regression analysis. All statistics were checked using a two-sided test, and statistical significance was viewed at *p* < 0.05. 

## 3. Results

### 3.1. Characteristics of Kindergarten Teachers

Kindergarten teachers aged between 17 and 54 (Mean ± SD: 33.01 ± 9.58) were more distributed at the age of 17–30 years (42.1%). Most teachers were female (98.2%); 70.1% of participants were married; 59.4% were from public kindergarten; 74.8% were from urban areas; and 44.1% have served as teachers for a decade or more years. About 5.5% of the participants had basic education; 57.7% had secondary education; and 36.8% had completed college education. We found that 13.9% of the participants were underweight and 7.2% were overweight. In addition, 67.9% of the participants were professionally trained (see Table 1).

### 3.2. Nutrition-Related Knowledge

Table 2 presents the average scores of nutrition-related knowledge of kindergarten teachers based on age, gender, marital status, residence, educational level, BMI, type of kindergarten, working time, and professional training of kindergarten teachers. The total score of nutrition-related knowledge was 53.00; the mean score of nutrition-related knowledge was found to be 28.74 (SD: 6.69), which was equivalent to 54.2% of the total score. The average scores of seven parts from Dimension 1 to Dimension 7 in Table 2 were as follows: 3.68 ± 1.25, 4.10 ± 1.50, 6.33 ± 2.63, 3.00 ± 0.98, 6.45 ± 1.66, 1.91 ± 0.86, and 3.28 ± 1.37. The lowest average score was in “Nutrient elements-related knowledge” and the highest average score was in “Dietary fiber-related knowledge.” Statistically significant differences were found between age, gender, marital status, type of residence, education level, type of kindergarten, working time, BMI, professional training of kindergarten teachers, and total nutrition-related knowledge scores (*p* < 0.05) (see Table 2).

Table 2 shows that the scores of participants aged more than 41 years old were the highest in each part, whereas the scores of participants aged 17–30 years old were the lowest, except for “Children’s nutrition-related knowledge”. The average scores of married participants were higher than those of unmarried participants. The average scores of unmarried were lower than the total average scores. The average scores of teachers living in urban areas were higher than those living in rural areas. The average scores of participants living in rural areas were lower than the total average scores. The average scores of participants with higher education were higher than those with basic education and secondary education, which were higher than the total average scores. The average scores of teachers from public kindergarten were higher than those of teachers in private kindergarten. The average scores of participants in public kindergarten were lower than the total average scores. The scores of participants who had worked for more than 10 years were the highest, whereas the scores of participants who had worked for less than three years were the lowest, except for “Dietary fiber-related knowledge”. The scores of teachers who were overweight were the highest, and the scores of teachers who were underweight were the lowest. The average scores of overweight teachers were higher than the total average scores. The scores of teachers who are not professionally trained kindergarten teachers were higher than those who are professionally trained. The average scores of untrained teachers were higher than the total average scores. The differences in “Vitamin-related knowledge” and “Nutrient elements-related knowledge” were statistically significant within their demographic profile, except for gender (*p* < 0.05) (see Table 2).

### 3.3. Nutrition Knowledge Learning-Related Attitudes

About 31.3% of the kindergarten teachers had “the confidence to do the childhood nutrition work well”. The scores of teachers with self-confidence were higher than those with no confidence. Only 9.9% participants were “satisfied with the childhood nutrition knowledge they already had”. The scores of teachers who were satisfied with current nutrition knowledge were higher than those were unsatisfied. About 97.7% of the kindergarten teachers were “willing to learn more about children-related nutrition knowledge”; 94.0% were “willing to attend more young childhood nutrition education and training”; and 96.2% were “willing to get information related to children’s nutrition knowledge through new media (micro blog, WeChat, QQ, and others)”. The scores of willing teachers on three willingness questions were higher than those who were unwilling. Statistically significant differences were found between “Are you willing to learn more about young childhood nutrition knowledge?” and “Are you willing to get information related to children’s nutrition knowledge through new media (micro-blog, WeChat, QQ, and others)?” for the total of nutrition-related knowledge scores (*p* > 0.05) (see Table 3).

### 3.4. Nutrition Knowledge Learning-Related Practices or Behaviors

Only 38.7% of the participants “participated in childhood nutrition knowledge courses or training”. The average scores of teachers who participated in childhood nutrition knowledge courses or training were higher than those without. About 80.1% participants paid attention to children’s nutrition knowledge. The average scores in teachers who paid attention to children’s nutrition knowledge were higher than those without. More than half of the participants (71.4%) have sometimes taken the initiative to promote children’s nutrition knowledge to their relatives or friends. The average scores in teachers who have always (3.3%) taken the initiative to promote children’s nutrition knowledge to their relatives or friends were higher than those who had often (19.7%), sometimes (71.4%), and never (5.6%) taken initiative to promote children’s nutrition knowledge. Most of the participants (81.9%) sometimes took the initiative to learn about children nutrition in their spare time. The frequency of teachers to take the initiative to learn about children nutrition in their spare time is as follows: always (3.3%), often (12.1%), sometimes (82.0%), and never (2.8%). Statistically significant differences were found in “Have you participated in childhood nutrition knowledge courses or training?”; “Have you paid attention to children’s nutrition knowledge?”; “Have you ever taken the initiative to promote children’s nutrition knowledge to your relatives or friends?”; and “Have you ever taken the initiative to learn about child nutrition in your spare time?” for the total of nutrition-related knowledge scores (*p* > 0.05) (see Table 4).

In our study, the approach employed by kindergarten teachers to acquire knowledge about children’s nutrition were through network (80.8%), TV or radio (57.7%), newspapers and magazines or books (53.0%), medical staff (46.5%), family members or relatives or friends (45.1%), and training institutions (28.8%).

### 3.5. Multiple Linear Regressions to Identify Factors That Affect Nutrition-Related Knowledge

In multiple linear regression models, a significant difference was found among age, type of residence, type of kindergarten, BMI, professional training of kindergarten teachers, behavior of having ever participated in childhood nutrition education knowledge courses or training, behavior of having ever paid attention to children’s nutrition knowledge, and nutrition-related knowledge among Chinese kindergarten teachers (Radj^2^ = 0.454, *p* < 0.001). Kindergarten teachers acquire more nutrition knowledge as they grow older. Kindergarten teachers who lived in urban areas had more knowledge of nutrition than those living in rural areas. Kindergarten teachers who came from public kindergartens had more knowledge of nutrition than those from private kindergarten schools. Teachers who were overweight had more knowledge of nutrition. Teachers who had not been professionally trained had more knowledge of nutrition than those have been trained professionally. Teachers who participated in childhood nutrition knowledge courses or training had more knowledge of nutrition than those without. Teachers who paid attention to children’s nutrition knowledge had more knowledge of nutrition than those who did not pay attention to the nutrition knowledge of children. There was no statistical significance between marital status, education level, working time, knowledge learning related attitude, knowledge getting related attitude, knowledge promoting related behavior, knowledge learning related behavior and nutrition-related knowledge among Chinese kindergarten teachers (see Table 5).

## 4. Discussion

The KAP model has been frequently applied in health-related behavior changes and public health. According to the basic principle of the KAP model, improving knowledge will change attitudes and behaviors to reduce the human and economic burden of diseases [27]. For example, a KAP study in Iran showed that increased nutritional knowledge among Iranian households was important in promoting their healthy eating [28]. This model emphasizes the beneficial influence of nutrition on health promotion, risk reduction, and disease management [29]. A prior study showed that time spent in kindergarten may be important for the timing of future public health approaches [30]. Therefore, kindergarten teachers are important leaders who can influence nutrition education of these children.

This survey showed that the teachers who participated had limited grasp of nutritional knowledge and the correct score of total nutrition-related knowledge among them was less than 60.0%. Knowledge may be a key factor to initiating changes in dietary behavior [31]. However, if preschool teachers lack sufficient nutrition background, they cannot provide support to effective prevention plans targeted at childhood obesity [24]. A study on caregiver behavior at mealtime among child-care in the US found that only 13.0% of early childhood teachers are familiar with recommended dietary guidelines or understand guidelines [25]; a recent study of nutrition-related knowledge among teaching staff in the US found that only 3.0% of teachers correctly answered at least four questions on nutrition knowledge, and 18.0% correctly answered at least three (all five nutrition questions on the survey) [24]. The correct rate of nutrition-related knowledge among kindergarten teachers in China is higher than those in other countries. A consideration in our study that needs to be kept while interpreting is that the content and standard of nutrition knowledge was broad in our study questions. This study also found low average scores in all domains, and the lowest score was “nutrient elements-related knowledge”. Results showed that participants with low scores on this domain were likely to lack nutrient elements-related knowledge in most of the nutrition knowledge areas. They might have also held misconceptions in a range of nutrient elements-related knowledge, which mainly included nutrient elements content, or species or functions in food in our survey. The low score of knowledge of nutrient elements-related is an issue that may impede teacher’s ability to consume a well-balanced diet, which will result in poor dietary intakes that could affect their healthy dietary guidance for students. Most teachers have a preliminary understanding of the knowledge of nutrition, but it was still in a relatively low level range. 

To our knowledge, this study is the first to examine the relationship between nutrition-related knowledge and other factors among kindergarten teachers, including nutrition knowledge learning-related attitudes, nutrition knowledge learning-related behaviors, and the demographic characteristics of respondents. By employing multiple linear regression models, we found that age, type of residence, type of kindergarten, BMI, professional training of kindergarten teachers, behavior of having ever participated in childhood nutrition education knowledge courses or training, and behavior of having ever paid attention to children’s nutrition knowledge are the factors that influence nutrition-related knowledge.

This study found that kindergarten teachers acquire more knowledge as they get older. This finding may have resulted from the rich experience among teachers. This study also found that kindergarten teachers from rural areas or those who teach in private kindergarten have the least amount of knowledge. Previous studies showed that some teachers felt that they lacked knowledge of healthy nutrition in low socioeconomic status kindergartens [32]. A similar research on food-related beliefs among elementary and middle schoolteachers in the countryside of the United States found that many teachers lacked nutrition knowledge concerning key foods [33]. This finding is attributed to the fact that the healthy diet policy of small rural schools has been significantly reduced compared with urban schools [34]. Previous studies determined that the nutrition knowledge is lower in teachers with professional training than those without training. The reason may due to the lack of nutritional courses include the professional training of kindergarten teachers. Further study is needed to establish the causes of this phenomenon. Previous findings show that overweight people were more likely to have health problems [24]. The occupational social environmental factors, such as usual work in bent and twisted postures, less physical activities, high screen time activities, skipping breakfast, and continuous exposure to a high level of noise, are associated with unhealthy behaviors [17,35,36]. In our study, overweight teachers had the highest nutrition knowledge among the three grades of BMI, which suggests that these participants had certain but insufficient knowledge of nutrition to change their unhealthy lifestyles. 

Total scores of nutrition-related knowledge are associated with nutrition knowledge-related behaviors. Kindergarten teachers who participated in childhood nutrition knowledge courses or training and those who focused on the nutrition knowledge of children have more knowledge. The current study revealed that most of the participants (61.5%) had not participated in childhood nutrition knowledge courses or training. A previous study also showed that only one-third of early childhood caregivers received workshop or in-service nutrition training [25]. Furthermore, the present study shows that the scores of teachers who participated in nutrition knowledge courses or training are higher than those who did not. These findings are in line with the previous American studies of Healthy Child Care intervention that explored the average score of teachers’ knowledge, which rose significantly from pre-intervention to post-intervention [37]. Data from the current study indicated that strengthening the early training of nutrition-related knowledge is necessary for all kindergarten teachers of the full coverage. Most kindergarten teachers in our study had positive behaviors concerning nutrition knowledge, particularly those with higher nutrition-related knowledge. To foster teachers’ active learning of nutritional knowledge, school-based nutritional programs would be needed, such as taste lessons in the Dutch language [38].

Our study found that most respondents were willing to obtain information related to the nutrition knowledge of children through new media (micro-blog, WeChat, QQ, and others), and respondents who were willing to acquire information through new media were more likely to have more nutrition-related knowledge than that held neutral and disapproval opinions. Furthermore, 80.8% of respondents were likely to obtain children-related nutrition knowledge from their network. Some previous studies showed that some new media—based training programs or intervention studies are effective and available [39,40,41]. Therefore, using media to spread knowledge to preschool teachers should be worthy of further study.

The findings may have useful public health significance. Research that focused on kindergarten teachers may play an important part in health promotion, education, and prevention of childhood obesity. Some suggestions exist for improving the nutrition-related knowledge of kindergarten teachers to improve and meet the health education demands of kindergarten children. First, training of nutrition-related knowledge, especially “nutrient elements-related knowledge”, should be strengthened among kindergarten teachers and should be part of the national educational curriculum. Second, with regards to kindergarten teachers who are younger, live in rural areas, are overweight, and work in private kindergartens, it would be beneficial to adopt corresponding protocols for screening and education by a nutritionist or dietitian. Third, the media network can be used as a new way to spread knowledge to kindergarten teachers.

This study has certain limitations. First, data obtained through a cross-sectional survey did not permit us to determine the causality. Second, only 222 participants were included in this study. The sample size was relatively small, which may have affected the accuracy and reliability of some results of this study. Therefore, the investigators received training on the quality control of data collection before the investigation. Third, “calcium-related knowledge” and “nutrient elements-related knowledge” in the questionnaire were only assessed by four items and three items, respectively, which may introduce information bias because of the participants possibly being unfamiliar with these questions. Moreover, the evaluation of nutrition-related knowledge, attitude, and behavior among kindergarten teachers was self-reported. This evaluation was subjective.

## 5. Conclusions

Kindergarten teachers have low levels of nutrition knowledge in Chongqing, China. Most teachers have a positive attitude about learning nutrition knowledge and have less training about nutritious knowledge. Furthermore, age, type of residence, type of kindergarten, BMI, professional training of kindergarten teachers, behavior of having ever participated in childhood nutrition education knowledge courses or training, and behavior of having ever paid attention to children’s nutrition knowledge were associated with nutrition-related knowledge among kindergarten teachers. This study used the KAP model to provide implications for interventions that can be designed for improving the nutrition-related knowledge among kindergarten teachers.

## Figures and Tables

**Table 1:** The demographic characteristics of preschool teachers (*n* = 222).

Variables	n	%
Age	217	
17–30 years	93	42.9
31–40 years	68	31.3
41 years	56	25.8
Gender	219	
Male	4	1.8
Female	215	98.2
Marital status	221	
Unmarried	66	29.9
Married	155	70.1
Type of residence	222	
Urban	166	74.8
Rural	56	25.2
Education level	220	
Basic education	12	5.5
Secondary education	127	57.7
Higher education	81	36.8
Type of kindergarten	219	
Public kindergarten	130	59.4
Private kindergarten	89	40.6
Working time	213	
Below three years	63	29.6
Three to ten years	56	26.3
Above ten years	94	44.1
BMI	209	
Underweight	29	13.9
Normal	165	78.9
Overweight	15	7.2
Professional training of kindergarten teachers	221	
Yes	150	67.9
No	71	32.1

**Table 2:** The average score of each dimension in different variables (mean, SD).

Variables	Dimensions							Total Score
	1	2	3	4	5	6	7	
Age (*n* = 217)								
17–30 years	3.33 ± 1.37 *	3.87 ± 1.46	5.28 ± 2.66 *	2.76 ± 0.96 *	5.97 ± 1.86 *	1.68 ± 0.86 *	3.23 ± 1.43	26.12 ± 6.94 *
31–40 years	3.75 ± 1.04 *	4.18 ± 1.49	6.76 ± 2.31 *	3.09 ± 1.08 *	6.46 ± 1.50 *	2.07 ± 0.88 *	3.16 ± 1.37	29.47 ± 5.91 *
≥41 years	4.16 ± 1.10 *	4.46 ± 1.53	7.57 ± 2.35 *	3.23 ± 0.80 *	7.13 ± 1.22 *	2.04 ± 0.78 *	3.50 ± 1.23	32.09 ± 5.52 *
Gender (*n* = 219)								
Male	4.75 ± 1.25	4.25 ± 1.25	7.25 ± 2.36	3.50 ± 1.00	6.75 ± 0.95	2.50 ± 0.57	3.75 ± 1.70	32.75 ± 4.03
Female	3.66 ± 1.24	4.10 ± 1.50	6.32 ± 2.64	2.99 ± 0.96	6.46 ± 1.61	1.89 ± 0.86	3.27 ± 1.37	28.68 ± 6.96
Marital status (*n* = 221)								
Unmarried	3.27 ± 1.23 *	3.85 ± 1.30	5.14 ± 2.57 *	2.76 ± 1.02 *	6.09 ± 1.87 *	1.70 ± 0.87 *	3.06 ± 1.43	25.86 ± 6.64 *
Married	3.85 ± 1.22 *	4.21 ± 1.58	6.82 ± 2.50 *	3.09 ± 0.94 *	6.59 ± 1.54 *	1.99 ± 0.85 *	3.38 ± 1.33	29.94 ± 6.36 *
Residence (*n* = 222)								
Urban	3.75 ± 1.28	4.20 ± 1.50	6.84 ± 2.55 *	3.15 ± 0.85 *	6.58 ± 1.52 *	2.04 ± 0.83 *	3.45 ± 1.36 *	30.01 ± 6.15 *
Rural	3.50 ± 1.14	3.79 ± 1.49	4.82 ± 2.29 *	2.54 ± 1.17 *	6.04 ± 1.98 *	1.52 ± 0.87 *^j^	2.77 ± 1.26 *	24.96 ± 6.84 *
Education level (*n* = 220)								
Basic	3.67 ± 1.15	3.83 ± 1.85	6.75 ± 2.45 *	2.25 ± 1.60 *	5.58 ± 2.06 *	1.50 ± 0.52 *	3.50 ± 0.52	27.08 ± 6.17 *
Secondary	3.65 ± 1.26	4.07 ± 1.41	5.82 ± 2.61 *	2.88 ± 0.94 *	6.26 ± 1.58 *	1.79 ± 0.85 *	3.12 ± 1.36	27.58 ± 6.58 *
Higher	3.72 ± 1.24	4.21 ± 1.61	7.05 ± 2.57 *	3.26 ± 0.83 *	6.80 ± 1.62 *	2.12 ± 0.88 *	3.48 ± 1.45	30.64 ± 6.55 *
Type of kindergarten (*n* = 219)								
Public kindergarten	3.77 ± 1.21	4.35 ± 1.47 *	6.92 ± 2.54 *	3.15 ± 0.94 *	6.68 ± 1.71 *	2.09 ± 0.86 *	3.44 ± 1.30 *	30.41 ± 6.61 *
Private kindergarten	3.52 ± 1.28	3.78 ± 1.46 *	5.46 ± 2.56 *	2.75 ± 0.99 *	6.16 ± 1.49 *	1.64 ± 0.80 *	3.04 ± 1.45 *	26.35 ± 6.17 *
Working lifetime (*n* = 213)								
<3 years	3.30 ± 1.29 *	3.97 ± 1.27	5.35 ± 2.45 *	2.78 ± 1.06	6.19 ± 1.58	1.63 ± 0.78 *	3.17 ± 1.33	26.40 ± 6.25 *
3–10 years	3.71 ± 1.31 *	3.98 ± 1.73	6.36 ± 2.86 *	2.95 ± 0.96	6.21 ± 1.87	2.00 ± 0.93 *	3.25 ± 1.61	28.46 ± 7.66 *
>10 years	3.95 ± 1.15 *	4.24 ± 1.50	6.80 ± 2.44 *	3.16 ± 0.93	6.74 ± 1.57	2.03 ± 0.87 *	3.37 ± 1.27	30.30 ± 6.10 *
BMI (*n* = 209)								
Low	3.31 ± 1.28 *	3.86 ± 1.43	4.48 ± 2.58 *	2.38 ± 1.17 *	5.72 ± 1.99 *	1.55 ± 0.73 *	3.00 ± 1.58	24.31 ± 6.93 *
Normal	3.69 ± 1.20 *	4.10 ± 1.53	6.45 ± 2.61 *	3.06 ± 0.92 *	6.47 ± 1.60 *	1.90 ± 0.89 *	3.25 ± 1.34	28.93 ± 6.53 *
Overweight	4.40 ± 1.24 *	4.80 ± 1.26	7.87 ± 1.24 *	3.40 ± 0.82 *	7.00 ± 1.55 *	2.40 ± 0.63 *	3.60 ± 1.24	33.47 ± 4.03 *
Professional training of kindergarten teachers (*n* = 221)								
Yes	3.61 ± 1.26	4.05 ± 1.46	5.94 ± 2.60 *	2.97 ± 0.91	6.38 ± 1.75	1.81 ± 0.87 *	3.20 ± 1.38	27.97 ± 6.52 *
No	3.85 ± 1.21	4.20 ± 1.60	7.15 ± 2.55 *	3.06 ± 1.12	6.56 ± 1.45	2.08 ± 0.82 *	3.45 ± 1.33	30.35 ± 6.84 *

Notes: (1) Dimension 1: Protein-related knowledge; Dimension 2: Fat-related knowledge; Dimension 3: Vitamin-related knowledge; Dimension 4: Calcium-related knowledge; Dimension 5: Dietary fiber-related knowledge; Dimension 6: Nutrient elements-related knowledge; Dimension 7: Children’s nutrition-related knowledge; (2) Abbreviations: SD, standard deviation; BMI, Body mass index; (3) *T*-tests, ANOVA and Mann–Whitney U tests were used to compare differences in continuous variables; (4) * statistical significance (*p* < 0.05).

**Table 3:** The average score of each dimension in nutrition knowledge-related attitudes.

Variables	*n* (%)	Total Score of Nutrition Knowledge (mean, SD)	*p*-Value
Do you have the confidence to do the childhood nutrition work well? (*n* = 211)			
Confident	66 (31.3)	29.24 ± 6.75	0.974
Neutral	100 (47.4)	29.01 ± 6.14	
No confident	45 (21.3)	29.04 ± 6.99	
Are you willing to learn more about children-related nutrition knowledge? (*n* = 216) *			
Willing	211 (97.7)	29.10 ± 6.52	0.029*
Neutral	3 (1.4)	21.00 ± 6.24	
Unwilling	2 (0.9)	21.50 ± 7.77	
Are you satisfied with the childhood nutrition knowledge you already have had? (*n* = 213)			
Satisfied	21 (9.9)	31.38 ± 5.63	0.095
Neutral	62 (29.1)	29.58 ± 6.92	
Unsatisfied	130 (61.0)	28.28 ± 6.56	
Are you willing to attend more young childhood nutrition education and training? (*n* = 216)			
Willing	203 (94.0)	29.07 ± 6.55	0.094
Neutral	6 (2.8)	29.17 ± 8.44	
Unwilling	7 (3.2)	24.29 ± 6.67	
Are you willing to get information related to children’s nutrition knowledge through new media (micro-blog, WeChat, QQ, and others)? (*n* = 212) *			
Willing	204 (96.2)	29.15 ± 6.53	0.039*
Neutral	6 (2.8)	23.17 ± 6.40	
Unwilling	2 (0.9)	23.00 ± 1.41	

Notes: (1) Abbreviations: SD, standard deviation; (3) ANOVA and Mann–Whitney U test were used to compare differences in continuous variables; (4) * statistical significance (*p* < 0.05).

**Table 4:** The average score of each dimension in nutrition knowledge-related behaviors.

Variables	N (%)	Total Score of Nutrition Knowledge (mean, SD)	*p*-Value
Have you ever participated in childhood nutrition education knowledge courses or training? (*n* = 217) *			
Yes	84 (38.7)	31.50 ± 6.04	<0.001*
No	133 (61.3)	27.32 ± 6.42	
Have you ever paid attention to children’s nutrition knowledge? (*n* = 212) *			
Yes	170 (80.2)	29.59 ± 6.47	<0.001*
No	42 (19.8)	25.29 ± 6.26	
Have you ever taken the initiative to promote children’s nutrition knowledge to your relatives or friends? (*n* = 213) *			
Never	12 (5.6)	29.00 ± 5.36	<0.001*
Sometimes	152 (71.4)	27.73 ± 6.58	
Often	42 (19.7)	31.76 ± 5.62	
Always	7 (3.3)	35.86 ± 3.71	
Have you ever taken the initiative to learn about child nutrition in your spare time? (*n* = 215) *			
Never	6 (2.8)	25.83 ± 4.40	0.001*
Sometimes	176 (81.9)	28.30 ± 6.63	
Often	26 (12.1)	31.81 ± 5.50	
Always	7 (3.3)	36.29 ± 4.11	

Notes: (1) Abbreviations: SD, standard deviation; (3) *T*-test and ANOVA were used to compare differences in continuous variables; (4) * statistical significance (*p* < 0.05).

**Table 5:** Multiple linear regressions to identify factors that affect nutrition-related knowledge.

Variables	*β*	*SE*	*T*	*p*
Age	2.242	0.770	2.913	0.004 *
Marital status	−1.288	1.283	−1.004	0.317
Type of residence	−3.856	0.997	−3.867	<0.001 *
Education level	0.806	0.871	0.925	0.356
Type of kindergarten	−2.355	0.975	−2.415	0.017 *
Working time	−0.804	0.773	−1.040	0.300
BMI	2.056	0.962	2.137	0.034 *
Whether it has been professionally trained	2.221	0.913	2.433	0.016 *
Knowledge Learning related Attitude	−1.884	1.664	−1.132	0.259
Knowledge Getting related Attitude	−2.740	1.546	−1.772	0.078
Knowledge Getting related Behavior	−1.872	0.945	−1.980	0.049 *
Knowledge Attending related Behavior	−3.659	1.102	−3.321	0.001 *
Knowledge Promoting related Behavior	1.648	0.879	1.876	0.063
Knowledge Learning related Behavior	0.025	0.951	0.027	0.979

Notes: (1) Knowledge Learning related Attitude: Are you willing to learn more about children-related nutrition knowledge; (2) Knowledge Getting related Attitude: Are you willing to get information related to children’s nutrition knowledge through new media (microblogging, WeChat, QQ, and others); (3) Knowledge Learning related Behavior: Whether have you participated in childhood nutrition education knowledge courses or training; (4) Knowledge Attending related Behavior: Whether have you paid attention to children’s nutrition knowledge; (5) Knowledge Promoting related Behavior: Have you ever taken the initiative to promote children’s nutrition knowledge to your relatives or friends; (6) Knowledge Learning related Behavior: Have you ever taken the initiative to learn about child nutrition in your spare time; (7) * statistical significance (*p* < 0.05).

## Supplementary Materials

The following are available online at http://www.mdpi.com/1660-4601/15/4/615/s1. Supplementary Materials S1: The contents of seven dimensions.
