# Supplementary material for: Nutrition-Related Knowledge, Attitudes, and Practices (KAP) among Kindergarten Teachers in Chongqing, China: A Cross-Sectional Survey
Source: Int J Environ Res Public Health. 2018 Mar 28;15(4):615. doi: 10.3390/ijerph15040615 (PMC5923657; doi:10.3390/ijerph15040615)
Supplement: Supplementary file 1 [file ijerph-15-00615-s001.pdf]

Supplement Materials S1. The specific contents of seven dimensions of nutrition-related knowledge

Dimension 1:Protein-related knowledge（**7 items**）
Protein is not stored in the human body, so it needs to be consumed every day.
Eggs and their products are a better source of protein than meat.
Protein in legumes belongs to high quality protein.
Protein can promote the growth and development of children.
Some of the amino acids are not synthesized by children themselves.
Protein is the primary nutrient for muscle energy.
The same equivalent carbohydrates and proteins provide the same heat value.
Dimension 2: Fat-related knowledge **(7 items)**
Appropriate lipids can improve children's immunity.
Red meat (such as beef, pork, etc.) all contains high saturated fatty acids.
Body fat plays an important role in the human body.
15%The energy provided by dietary fat should not exceed 15% of the daily energy supply.
Reducing the amount of fat and fat meat in the diet is good for your health.
Proper supplementation of lipids can improve the absorption of vitamin A among children.
Lipids can promote the development of children's intelligence.
Dimension 3: Vitamin-related knowledge **(13 items)**
People who participate in sports should be supplemented with a certain amount of vitamin.
Vitamins are also a good class of energy sources.
In general, fat soluble vitamin intake is not the risk of poisoning.
Green leaf vegetables and carrots are rich in vitamin A.
People can produce vitamin D through the sun.
Vitamin E is widely used in food, and it is not lacking for the human body.
Green leaves, beans and eggs are rich in vitamin B1.
Spinach, potatoes, oranges, and strawberries all contain more vitamin C.
Adequate intake of vitamin A can prevent night blindness, while also reducing the generation of free radicals.
Supplementing calcium with vitamin D, the effect is better.
Vitamin E can not only resist oxidation, delay aging, but also promote sex hormone secretion
Vitamin C is also called ascorbic acid.
“Beriberi” is a typical symptom of vitamin B2 deficiency.
Dimension 4: Calcium-related knowledge **(4 items)**
Carbonated drinks have a negative effect on calcium absorption.
Cauliflower is a vegetable rich in calcium.
Milk and dairy products are good sources of calcium.
Children should pay attention to the intake of sufficient calcium to meet the needs of growth and development.
Dimension 5: Dietary fiber-related knowledge **(9 items)**
Eating beans and soy products is good for health.
Eating a lot of fruit and vegetables is very good for health.
Eating more sugar is good for your health.

Eating different kinds of food is good for health.
Milk and dairy products are good for health.
Eating a large number of staple foods, such as rice, is unhealthy.
Eating beans and soy products is good for health.
A vegetarian diet increases the risk of iron deficiency.
Salt is one of the basic components of a healthy diet.
Dimension 6: Nutrient elements-related knowledge **(3 items)**
Meat and eggs are rich in zinc.
Potatoes, spinach, and bananas contain more potassium.
Diabetes causes more sugar to eat.
Dimension 7: Children's nutrition-related knowledge **(10 items)**
The number of "average dietary pagoda" of Chinese Residents.
Energy supply ratio of three meals at early and late evening.
How is the best nutritional supplement for children.
Optimal daily average salt intake for children (3-6 years old).
The optimal daily average consumption of oil and oil in children.
Children drink a small amount of water every day.
Daily average daily intake of vegetables in children.
Optimal daily fruit intake for children.
The best weekly exercise volume (unit: hour) for preschool children.
The optimum intake of calcium daily for children.
